# Three-Dimensional Crop Phenotyping for Crop Protection: Reconstruction Routes, Decision Pathways, and Digital-Twin Maturity

**DOI:** 10.3390/plants15131992

**Published:** 2026-06-27

**Authors:** Fanguo Zeng, Lin Yuan, Ouguan Xu, Chong Li

**Affiliations:** 1 School of Computer Science and Technology, Zhejiang University of Water Resources and Electric Power, Hangzhou 310018, China; zengfg@zuwe.edu.cn (F.Z.); yuanl@zjweu.edu.cn (L.Y.); xuog@zuwe.edu.cn (O.X.); 2 Key Laboratory of Carbon Sequestration and Emission Reduction in Agriculture and Forestry of Zhejiang Province, School of Environmental and Resources Science, Zhejiang A&F University, Hangzhou 311300, China

**Keywords:** 3D crop phenotyping, crop protection, 3D reconstruction, digital twin maturity, decision support

## Abstract

Three-dimensional (3D) crop phenotyping is increasingly used to capture crop structure, but its value for crop protection is conditional rather than automatic. 3D approaches are operationally justified only when reconstructed geometry adds decision-relevant information beyond simpler 2D, spectral, scalar, or conventional baselines. This review examines 3D crop phenotyping through a reconstruction–trait–task–maturity framework for crop protection and synthesizes evidence across disease assessment, pest and stress interpretation, pesticide dose adjustment, spray deposition, weed-target perception, protection-oriented breeding, and digital-twin development. The literature is organized through four connected lenses: reconstruction routes that generate crop geometry, 3D traits that may alter protection reasoning, decision pathways that link traits to intervention variables, and maturity levels that distinguish static 3D models, validated phenotypic traits, process-coupled systems, protection outputs, and outcome-updated decision twins. The strongest decision-facing evidence currently comes from canopy-based dose adjustment, deposition prediction, drift reduction, and related spraying applications in which 3D traits are linked to intervention variables and field-facing comparators. Disease, stress, and architecture-aware modelling provide important but more heterogeneous evidence, while many point-cloud datasets, segmentation pipelines, neural reconstruction methods, and agricultural digital-twin frameworks remain upstream of practical crop-protection decisions because they do not yet connect 3D measurements to validated protection labels, comparator baselines, decision thresholds, intervention outputs, or outcome updating. A central conclusion is that high-fidelity 3D representation should not be conflated with decision-twin maturity. Protection-oriented digital twins require explicit coupling among synchronized crop geometry, functional or epidemiological models, decision rules, and recorded field outcomes. This review therefore identifies the evidence and reporting priorities needed to move 3D crop phenotyping toward validated, deployment-oriented, and feedback-aware crop-protection support.

## 1. Introduction

Crop protection is increasingly shaped by the need for earlier risk detection, more precise intervention, reduced pesticide inputs, and more resilient breeding decisions under heterogeneous field conditions. Many of these challenges are inherently spatial and structural. Pathogen dispersal is influenced by canopy architecture and microclimate [1]; pesticide deposition depends on target geometry and occlusion; stress symptoms vary with organ position and growth stage; and pest or disease scouting depends on the three-dimensional arrangement of leaves, stems, fruits, and canopy gaps. Although two-dimensional (2D) imagery has substantially advanced crop monitoring, it often compresses this structural complexity into projected appearance. Three-dimensional (3D) crop phenotyping therefore provides a complementary route for making crop geometry measurable, comparable, and, in principle, actionable.

Recent plant-phenotyping research has advanced 3D reconstruction through classical multi-view methods, LiDAR and point-cloud pipelines, and neural representations such as Neural Radiance Fields (NeRF) and 3D Gaussian Splatting (3DGS) [2,3]. Field systems, including backpack LiDAR and CropQuant-3D, have demonstrated that plot-scale wheat architecture can be measured at scale [4]. At the same time, benchmark datasets and organ-level segmentation methods are improving the reproducibility of 3D perception and trait extraction, including annotated wheat point-cloud resources for part segmentation under contrasting water conditions [5,6,7,8]. In parallel, developments in smart farming and functional-structural plant modelling have renewed interest in digital twins [9,10,11]. Together, these developments create an opportunity to reconsider the role of 3D crop phenotyping in crop protection.

Although 3D crop reconstruction and agricultural digital twins have been widely reviewed, the decision pathway that links reconstructed structures to crop-protection outputs remains less explicit. This review therefore treats 3D crop phenotyping as a chain of evidence rather than as a reconstruction catalogue. The core question is whether a 3D trait changes a protection task—such as severity assessment, pest or stress interpretation, spray or dose decision, risk modelling, or breeding choice—relative to the information available from simpler 2D, spectral, scalar, or conventional baselines. This framing deliberately separates geometric fidelity from decision evidence: a reconstruction is interpreted as protection-relevant only when it is connected to validated traits, biological or physical process models, intervention variables, or outcome records.

This review makes four operational contributions. First, it defines a reconstruction–trait–task–maturity chain that can be used to judge whether a 3D method remains an enabling measurement layer or has reached protection relevance. Second, it classifies evidence by protection task—including severity assessment, disease-process modelling, spray dosing, deposition, scouting, robotic action, breeding, and feedback—so that sensor novelty is not mistaken for agronomic utility. Third, it uses an L0–L5 maturity ladder to separate static 3D models, validated phenotypic traits, process-coupled models, intervention outputs, and outcome-updated decision systems. Finally, it proposes reporting criteria centred on the crop target, 3D trait, comparator, decision output, validation context, and feedback status.

**Figure 1:**
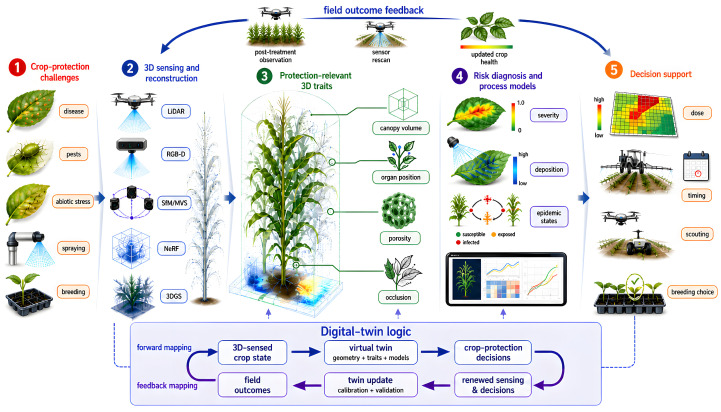
3D crop phenotyping for crop-protection decisions. The figure presents 3D crop phenotyping as a trait-to-decision infrastructure that links crop-protection challenges, upstream 3D sensing and reconstruction, protection-relevant traits, risk diagnosis and process models, decision-support outputs, digital-twin logic, and field outcome feedback. Colored connecting lines indicate the main information pathways among sensing, traits, decision support, and feedback.

**Figure 2:**
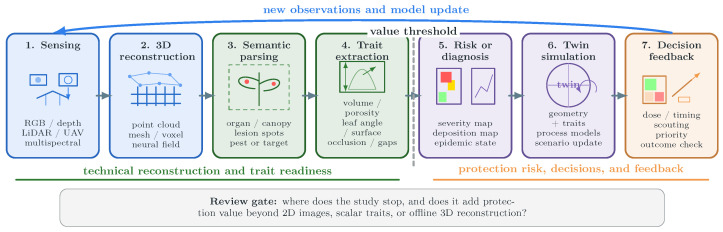
Sensing-to-decision pipeline for 3D-enabled crop protection. The figure separates technical reconstruction and trait-readiness stages from protection-risk, twin-simulation, decision, and feedback stages, emphasizing that 3D reconstruction gains crop-protection value when extracted traits are linked to diagnosis, process models, intervention outputs, validation, and renewed observations. Arrows show information flow through the pipeline, colored squares denote major processing or decision stages, and dots indicate feedback or update points.

**Figure 3:**
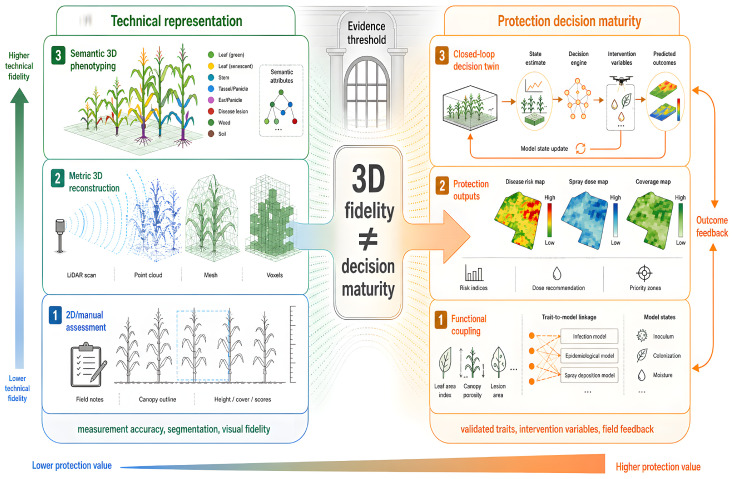
Maturity-positioning framework linking technical 3D representation to protection decision maturity. The figure distinguishes increasing technical fidelity in 2D/manual assessment, metric 3D reconstruction, and semantic 3D phenotyping from the evidence threshold required for functional coupling, protection outputs, and closed-loop decision-twin logic. It emphasizes that 3D fidelity is useful but not sufficient: crop-protection value emerges when validated traits are connected to intervention variables, protection maps, model updating, and outcome feedback.

To make this argument operational, the review uses a figure–table architecture rather than a catalogue of technologies. Figure 1 defines the overall logic linking crop-protection challenges to 3D traits, digital-twin reasoning, decisions, and feedback. Figure 2 extends this logic into a sensing-to-decision pipeline, while Figure 3 positions current methods within an evidence architecture that separates technical representation from protection-decision maturity. The figures are conceptual organizers, not quantitative evidence maps; their role is to make the classification criteria and weak links visible before the evidence tables are interpreted. Table 1 first summarizes the evidence-mapping logic used for study classification. Four later tables provide the evaluative backbone of the review. Table 2 first organizes the reconstruction-method landscape and identifies what each method can contribute to protection-oriented phenotyping. Table 3 then shifts the emphasis from methods to tasks, showing where 3D traits can change severity assessment, spraying, scouting, modelling, or breeding outputs. Table 4 provides an evaluative ladder that distinguishes static 3D models from phenotypic, functional, protection, and decision twins. Finally, Table 5 converts the remaining evidence gaps into a reporting and research agenda. Together, these elements define the review architecture: reconstruction methods are interpreted through traits, traits through protection tasks, digital-twin claims through maturity criteria, and research gaps through missing comparator, decision, validation, or feedback evidence.

## 2. Review Scope and Evidence Classification

This paper is a critical narrative review with structured evidence classification. The narrative format was chosen because the relevant evidence is distributed across plant phenotyping, plant pathology, precision spraying, robotics, functional-structural plant modelling (FSPM), digital agriculture, and smart-farming literatures, where study designs, crops, sensors, validation targets, and decision outputs are too heterogeneous for a single quantitative synthesis. The aim was therefore not to estimate pooled effect sizes, but to classify how strongly different types of 3D evidence support protection decisions. To reduce selection bias within this narrative format, the review used explicit search, screening, and classification rules; retained evidence that both supported and constrained the decision-oriented framework; and interpreted conflicting, negative, or weakly validated findings as maturity limitations rather than as grounds for exclusion.

### Search and Selection Strategy

Literature was identified through iterative searches in Web of Science, Scopus, Google Scholar, IEEE Xplore, ScienceDirect, SpringerLink, MDPI, Frontiers, and publisher pages for precision-agriculture and plant-phenotyping journals, with backward and forward citation checking of anchor papers. Searches were updated during manuscript preparation through May 2026. Search terms were combined across four blocks: (i) 3D sensing and reconstruction terms, including “3D crop phenotyping”, “plant point cloud”, “LiDAR”, “RGB-D”, “stereo”, “structure from motion”, “multi-view stereo”, “NeRF”, and “3D Gaussian Splatting”; (ii) crop-protection terms, including “plant disease”, “pest”, “weed”, “stress”, “spray deposition”, “variable-rate application”, “pesticide dose”, and “crop protection”; (iii) decision and validation terms, including “severity”, “threshold”, “decision support”, “prescription map”, “robotic”, “field validation”, and “feedback”; and (iv) digital-agriculture terms, including “digital twin”, “functional-structural plant model”, “smart farming”, and “precision agriculture”.

Studies were included when they met at least one of three criteria: direct crop-protection relevance, such as disease severity, pest or weed targeting, stress interpretation, spray dosing, deposition, drift, scouting, or protection-oriented breeding; reusable 3D phenotyping infrastructure, such as crop-specific reconstruction, segmentation, benchmark datasets, organ-level point clouds, or temporal 3D traits that could support protection tasks; or digital-twin or FSPM relevance, where 3D crop structure was coupled to biological, physical, intervention, or decision-support models. Studies were excluded from high-maturity interpretation when they presented generic 3D reconstruction, visualization, or digital-twin language without crop-specific validation, protection labels, trait extraction, process coupling, intervention output, or feedback. Because the review is not a PRISMA-style systematic review, it does not claim exhaustive coverage of every 3D reconstruction or disease-imaging paper; instead, it aims to synthesize the evidence most relevant to the pathway from 3D measurement to crop-protection decision. To make the selective scope explicit, studies were not treated as stronger merely because they used more advanced sensors or algorithms. Evidence was weighted upward only when it reported a protection-relevant output, a comparator or baseline, a field or biologically meaningful validation context, or a link to intervention variables. Evidence was weighted downward when it reported only reconstruction quality, visualization, laboratory-only feasibility, untested transfer across field conditions, or a protection claim without labels, thresholds, or outcome linkage. Where findings conflicted across sensors, crops, or platforms, the synthesis emphasized the boundary conditions under which each claim was made rather than presenting the most favourable result as general.

All recent or online-first references, especially 2025–2026 studies, were treated as evidence requiring bibliographic verification before submission. In the synthesis below, recently published studies are used conservatively: they strengthen evidence only when the bibliographic record includes a traceable journal venue and DOI and when the reported output can be connected to a trait, comparator, decision variable, or validation context.

Evidence was classified by function rather than by sensor type alone. First, studies were assigned to review roles, including framework anchors, enabling reconstruction or segmentation methods, direct application evidence, digital-twin or FSPM bridges, benchmark or dataset infrastructure, and future-agenda sources. Second, each study was interpreted according to maturity: static reconstruction, validated phenotypic trait, process-model coupling, protection-risk or intervention output, and decision feedback. Third, protection relevance was judged by whether the 3D information changed a measurable task output, such as a disease-severity score, a canopy-volume dose variable, a deposition prediction, a spray command, a risk-model state, a scouting priority, or a breeding trait. This classification is intentionally conservative. A high-fidelity 3D model is treated as enabling evidence unless it is linked to validated traits, biological processes, intervention rules, or field outcomes. Negative or inconclusive signals—for example, lack of a 2D comparator, unstable field transfer, missing protection labels, or absence of treatment thresholds—were retained as evidence of a gap and were used to limit the maturity assigned to that study type. The same principle guides the terminology of the review: digital-twin-ready or feedback-aware language is used when the evidence supports component readiness, whereas the term decision twin is reserved for systems that connect sensing, prediction, recommendation, and outcome updating.

Table 1 summarizes the classification logic; the later task and maturity tables then apply this logic to specific evidence pathways rather than attempting to make each table cell a complete annotated bibliography.

**Table 1:** Evidence-mapping logic used for study classification. The table makes explicit how representative studies were interpreted before being placed in the later reconstruction, task, and maturity tables.

Evidence Role	Minimum Inclusion Signal	Decision-Facing Test	Typical Maturity Range	Representative Examples
Reconstruction or benchmark infrastructure	Crop-specific 3D scenes, point clouds, datasets, or segmentation outputs.	Support reusable traits or labels for protection tasks.	L0–L2	Reconstruction surveys, CropQuant-3D, Crops3D, Pheno4D, Wheat3D PartNet, and organ segmentation [2,4,5,8].
Direct protection application	3D or depth-derived information is linked to protection outputs.	Change severity scoring, dose, deposition, scouting priority, robotic target, or genotype interpretation.	L2–L4	Disease-severity imaging, LiDAR disease or stress studies, VRA, deposition modelling, and robotic weeding [12,13,14,15].
Process or digital-twin bridge	3D traits are coupled to FSPM, epidemiological, spray-transport, risk, or smart-farming models.	Explain, predict, recommend, or update a protection-relevant state.	L3–L5 components	Septo3D, FSPM disease modelling, digital-twin frameworks, and deposition prediction [9,16,17,18].
Future-agenda evidence	Studies expose missing labels, baselines, field transfer, interoperability, or feedback.	Identify a missing link in the trait-to-decision pathway.	Cross-level gap evidence	Benchmark datasets without protection labels, field-transfer studies, and open-loop VRA or digital-twin components.

The maturity range is not a quality score. It identifies the highest decision function demonstrated by the evidence, not the sophistication of the sensor or reconstruction method.

Accordingly, generic 3D reconstruction studies or agricultural digital-twin claims are not treated as high-maturity protection evidence when they lack crop-specific validation, protection labels, or decision linkage. Conversely, field and platform studies with modest reconstruction novelty may have high protection relevance when they connect canopy structure to dose reduction, deposition, efficacy, disease severity, or decision outputs. In assessing the active evidence base, particular attention was given to whether each study reported the crop and protection target, the 3D or depth-derived trait, the comparator or baseline, the validation environment, and the extent to which the output could support an intervention or feedback loop. This evidence logic supports the figures and tables below and helps prevent digital-twin terminology from substituting for demonstrated synchronization, prediction, recommendation, or feedback.

This classification also defines the value of the review for readers. Rather than leaving maturity to be inferred from heterogeneous methods, the manuscript makes the review criteria explicit: what is measured, what biological or physical mechanism it represents, what decision variable it can change, and what evidence is still missing before operational use. The result is a synthesis that is deliberately selective in its claims while still making visible where 3D crop phenotyping is already close to practical protection value.

## 3. Conceptual Framework: From 3D Geometry to Protection Decisions

The literature is organized here as a sensing-to-decision pipeline. The first layer captures crop structure through RGB, depth, multispectral, LiDAR, or multi-view imagery. The second layer reconstructs geometry as meshes, point clouds, voxel models, neural scene representations, or functional–structural plant models. The third layer converts geometry into phenotypic traits, including canopy volume, leaf-angle distribution, organ position, plant height, occlusion, gap fraction, surface area, disease-lesion topology, or target density. The fourth layer links these traits to protection tasks, such as disease-severity estimation, disease-spread modelling, pest-habitat inference, abiotic-stress assessment, pesticide-dose adjustment, spray deposition, and protection-oriented breeding. The final layer translates trait and model outputs into management or breeding decisions.

This framework distinguishes 3D representation from 3D-enabled decision support. A reconstructed crop scene becomes useful for crop protection only when its geometric or functional traits provide information that cannot be obtained reliably from a 2D image, or when they improve robustness, interpretability, or transferability. For example, canopy volume and leaf-area distribution can directly affect pesticide dosage and deposition [12,19,20], whereas architecture-aware disease models can be used to test how canopy structure modifies pathogen progress [16,17,21]. Laser- and sensor-guided orchard or vineyard sprayers further show how canopy detection can move 3D measurement toward actuator-level dose and coverage decisions, with complementary PWM, ultrasonic profile-sensing, and seasonal canopy-adjustment studies reinforcing this geometry-to-application link [22,23,24,25,26,27]. By contrast, a visually impressive 3D model without trait validation or intervention linkage remains an upstream measurement product. Figure 2 translates this principle into a pipeline for interpreting the following sections.

## 4. 3D Reconstruction Foundations for Crop Protection

### 4.1. Sensing, Reconstruction, and Benchmark Infrastructure

The 3D reconstruction foundation for crop protection involves both metric reconstruction and semantic interpretation. General reviews of 3D phenotyping emphasize that crop geometry can be measured through several routes, including photogrammetry, laser scanning, depth cameras, structured light, and multi-view systems [2]. LiDAR and point-cloud pipelines are now sufficiently mature to quantify plant height, canopy envelope, structural variation, canopy density, canopy volume, and organ-scale traits under controlled horticultural and field conditions [4,7,28,29]. Studies using multi-view stereo, structure-from-motion, RGB-D, stereo vision, and structured light further indicate that low-cost or close-range optical methods can recover organ- or row-scale geometry, although their robustness remains strongly dependent on acquisition conditions. The most important operational constraints are not only reconstruction accuracy under ideal capture, but whether wind, illumination change, specular or texture-poor organs, dense canopy occlusion, and platform motion degrade the traits that a protection decision would use. Datasets such as Pheno4D, Crops3D, and Wheat3D PartNet provide infrastructure for evaluating 3D perception, temporal point-cloud analysis, realistic crop segmentation, and organ-part segmentation in crop point clouds, while RGB-D dataset surveys clarify data, ground-truth, and benchmarking requirements for robotic site-specific operations [5,6,8,30]. Such resources are important because crop-protection systems must operate under occlusion, growth dynamics, species diversity, field variability, and task-specific annotation constraints.

### 4.2. Neural Reconstruction and Protection-Readiness

At the methodological frontier, neural reconstruction methods such as NeRF and 3DGS can provide high-fidelity plant geometry and appearance [3]. Their crop-protection value, however, should be assessed in terms of downstream utility rather than visual realism alone. Compared with terrestrial or mobile LiDAR, NeRF and 3DGS are more dependent on multi-view image consistency: wind-driven leaf motion can violate the static-scene assumption, variable ambient lighting can change apparent colour and view-dependent reflectance, and heavy occlusion can leave disease-relevant inner canopy organs weakly constrained. LiDAR is not immune to occlusion or registration error, but it provides direct metric range measurements and is less dependent on texture or illumination, which makes it a stronger backbone when the decision variable is canopy volume, porosity, height, or target geometry under variable field light. The key questions are therefore whether neural representations improve the measurement of disease-relevant organs, support synthetic data generation for scouting models, enable more accurate spray-target reconstruction, or improve temporal consistency for stress monitoring after these field constraints are considered. For this reason, neural reconstruction is treated in this review as a promising enabling technology rather than as direct evidence of a protection digital twin.

Recent crop-specific neural reconstruction studies illustrate the rapid movement from generic scene reconstruction toward plant-level structural representation. Examples include NeRF-based high-fidelity plant reconstruction [31], few-shot cross-species plant-instance point-cloud reconstruction with PlantSegNeRF [32], and object-centric 3D Gaussian splatting for strawberry plant reconstruction and phenotyping [33]. These studies enrich the methodological roadmap, but they also clarify the evaluation criterion used in this review: neural 3D representations should be promoted from L0–L1 reconstruction evidence to protection evidence only when they yield validated traits, disease or stress indicators, spray targets, or decision-relevant model states. Table 2 therefore summarizes the main reconstruction-method classes as enabling layers for crop-protection phenotyping, rather than as substitutes for protection decisions.

**Table 2:** Review-oriented classification of 3D reconstruction routes for crop-protection phenotyping. The table separates technical capability from the additional evidence needed before a method can support protection decisions.

Method Class	Typical Data and Output	Main Crop-Scene Trade-Off	Protection-Relevant Use	Evidence Status for This Review
Part 1				
SfM/MVS photogrammetry	Multi-view RGB from ground, gantry, or UAV platforms; point clouds, meshes, and canopy surface models.	Low-cost and visually detailed, but trait extraction is sensitive to texture, illumination shifts, wind-induced leaf motion, occlusion, and scale consistency; these errors can propagate into canopy volume, lesion position, or spray-accessibility estimates.	Canopy height, volume, lodging, and architecture proxies for disease, pest, stress, or spray contexts.	Enabling L1 evidence unless traits are validated against protection outcomes [2].
Terrestrial or mobile LiDAR	Static TLS, backpack LiDAR, ground vehicles, and robotic platforms; dense metric point clouds and registered crop structure.	Accurate, metric, and texture-independent under variable light, but still affected by sensor cost, occlusion shadows, registration complexity, scanning geometry, and platform logistics.	Canopy structure, plant height, porosity, organ position, canopy density, canopy volume, and field robustness.	Strongest measurement backbone for L1–L2 phenotyping layers when geometric traits are the decision input; protection value increases when traits are linked to dose, disease, or stress decisions [4,7,28,29].
UAV LiDAR and aerial 3D mapping	UAV LiDAR, UAV photogrammetry, and aerial point clouds; canopy-height models and plot-scale structural maps.	Provides field-scale spatial coverage, but usually has limited organ resolution and remains constrained by occlusion, flight regulation, and sensor cost.	Plot-scale scouting, biomass or stress heterogeneity, and management-zone interpretation.	Useful for spatial prioritization, but often upstream of direct treatment decisions [34].
RGB-D, depth, and stereo vision	Depth cameras, stereo images, mobile robots, AR or handheld devices; depth maps and local point clouds.	Low-cost and compatible with scouting or robotics, but limited by sunlight sensitivity, range, calibration drift, partial-view geometry, and moving-canopy artefacts.	Organ localization, disease-severity imaging, depth-assisted masks, stereo–thermal detection, and robot-localized targets.	Moves toward L2 when task labels are validated; decision maturity requires treatment thresholds or action outputs [35,36,37].
Structured-light and high-resolution close-range scanning	Projected-light systems and controlled close-range imaging; high-resolution organ surfaces or lesion-scale geometry.	Precise morphology and surface measurement, but mostly controlled-environment and low-throughput.	Validation of organ morphology, fruit surfaces, or lesion geometry.	Mainly laboratory or reference evidence rather than operational crop-protection infrastructure [2].
Part 2				
Temporal point-cloud datasets and benchmarks	Repeated 3D scans, annotated point clouds, synthetic or realistic datasets; semantic labels and benchmark splits.	Improve reproducibility and cross-method comparison, but protection labels and field variability are often missing.	Growth normalization, segmentation benchmarking, and the infrastructure needed for disease, stress, or spray-accessibility labels.	Critical benchmarking infrastructure; Wheat3D PartNet illustrates progress toward annotated part segmentation under contrasting water regimes [5,6,8].
Semantic and organ-level point-cloud learning	LiDAR or reconstructed point clouds with organ labels; segmented organs, instances, skeletons, and organ-level traits.	Converts geometry into biological units, but annotation burden, transfer across crops, and occlusion robustness remain limiting.	Organ-localized disease scoring, spray targeting, architecture-aware ideotype selection, and stress-related part analysis.	Important L2 bridge; still requires validation beyond segmentation metrics to support protection decisions [7,8,38].
Neural radiance fields and 3D Gaussian Splatting	Multi-view images or video; implicit or explicit neural scene representations, rendered 3D crop scenes.	High-fidelity appearance and view synthesis, but field trait extraction is vulnerable to moving leaves, changing illumination, occluded organs, uncertain scale, training/runtime cost, dynamics, and limited field scalability compared with direct-range LiDAR.	Frontier reconstruction, synthetic scene generation, and potential digital-crop visualization.	Usually L0–L1 unless validated metric traits, biological states, or decisions are extracted under field constraints [3,31,32,33].
Multimodal 3D fusion	RGB, LiDAR or depth, spectral, thermal, weather, and environmental data; geometry plus functional or physiological state variables.	Better represents structural, physiological, and environmental risk, but requires calibration, synchronization, data standards, and causal interpretation.	Disease, stress, microclimate, and protection-risk inference where geometry alone is insufficient.	Most relevant for protection-twin development, but evidence should report explicit task outputs and decision comparators [37].

Part 1 summarizes core sensing and metric reconstruction routes. In this review, a reconstruction method is not treated as protection evidence unless it supports a validated trait, risk state, intervention variable, or decision-support input. Part 2 summarizes benchmark, semantic-learning, neural-reconstruction, and multimodal-fusion routes. Visual or geometric fidelity is treated as enabling evidence, not as protection value by itself.

Overall, this method landscape establishes what can be measured, but not yet what should be done with the measurement. The evidence is therefore read along two axes: technical capability and agronomic utility. Technical capability concerns reconstruction fidelity, semantic resolution, temporal consistency, and robustness under occlusion or field variability; agronomic utility concerns whether the resulting trait changes a disease score, spray variable, risk state, scouting priority, breeding target, or management recommendation. The operational threshold for adopting 3D is therefore higher than the threshold for demonstrating 3D reconstruction. Conventional 2D imagery, spectral indices, or scalar crop descriptors may be sufficient when symptoms are surface-visible, targets are sparse, or management decisions do not depend on canopy depth, organ position, porosity, volume, or occlusion. Full 3D deployment is better justified when the additional cost, computation, calibration, and field logistics are offset by a measurable improvement in a decision variable—for example dose adjustment, deposition prediction, organ-localized severity, target exposure, or architecture-aware disease risk. Having established the main sensing and reconstruction routes, the next section considers the protection tasks for which 3D traits appear most likely to alter assessment, prediction, or intervention beyond 2D imagery, scalar crop descriptors, or generic reconstruction benchmarks.

## 5. Protection-Relevant 3D Traits and Applications

### 5.1. Spraying, Deposition, and Intervention Variables

The clearest direct evidence for crop-protection value appears when 3D geometry or canopy structure is mapped to an intervention variable rather than used solely as a descriptive trait. Precision-spraying studies illustrate a recurring decision chain: canopy volume, leaf area, target density, or porosity is estimated; the trait is translated into dose, deposition, drift, airflow, or prescription-map output; and the output is evaluated against coverage, efficacy, input reduction, or off-target loss. DOSA3D provides a clear anchor for this pathway by estimating leaf area index, spraying efficiency, and pest or disease targets to adjust pesticide doses in fruit and grapevine orchards, with reported pesticide savings of up to 53% in fruit trees and 60% in vineyards while maintaining crop health [12]. Earlier orchard, vineyard, and nursery systems established the same logic with laser or ultrasonic canopy sensing, where tree, vine, or plant structure was used to synchronize nozzle output, reduce spray volume, maintain pest or disease control, maintain deposition, or reduce drift and off-target losses [19,20,39,40,41,42].

A similar decision-facing logic is evident in independent field and platform studies. UAV LiDAR-derived cotton canopy volumes reduced spray volume by 43.37% while maintaining biological efficacy [13]; LiDAR/RGB wheat biomass estimation has been linked to prescription-map interpretation and PWM-controlled UAV variable spraying [43]; voxelized peach-tree canopy point clouds predicted pesticide deposition with best-case $R^{2}$ values of 0.80–0.86 across fan speeds [18]; and a disease-aware fruit-tree sprayer fused canopy volume with spot detection to reduce dosage relative to both conventional and volume-only variable-rate spraying while maintaining pear-rust control efficacy [44]. Recent orchard systems move this linkage closer to real-time control by using laser-sensed canopy characteristics to regulate air and liquid output, maintaining canopy-edge air velocity at 3–5 m s^−1^ while reducing ground losses by 86.95% and airborne drift by 89.98% relative to conventional application [45].

A broader group of field studies extends this branch through UAV-SfM canopy characterization, porosity-informed droplet-penetration models, LiDAR-based canopy-volume sprayers, canopy-volume-based UAV spraying, air-assisted deposition analysis, laser-guided or sensor-based orchard spraying, PWM and ultrasonic profile-controlled spraying, UAV downwash or drift analysis, and intelligent-sprayer disease or insect management in orchards and vineyards [22,23,24,25,26,27,29,46,47,48,49,50,51,52,53,54]. Older laser-guided and ultrasonic VRA studies remain important because they document the transition from canopy detection to actuator-level spray control and environmental-loss reduction rather than only post hoc trait measurement [39,40,41]. This evidence stream is strong relative to other protection tasks because many studies report an intervention variable and a field-facing comparator, such as conventional volume, volume-only variable-rate spraying, deposition, efficacy, drift, or off-target loss. It should still be interpreted as partial protection-twin evidence: geometry is linked to decision variables and field performance, but outcome-updated prescriptions and closed-loop learning are usually absent.

### 5.2. Disease Severity, Symptom Localization, and Architecture-Aware Modelling

Disease modelling provides a second pathway in which 3D structure is valuable because it changes the interpretation of biological risk. Septo3D couples a 3D virtual wheat plant with a Septoria tritici epidemic model to investigate plant–pathogen interactions linked to canopy architecture, and coupled virtual wheat–Septoria modelling further shows how sowing-density-driven canopy architecture and inter-plant variability can influence epidemic simulation [16,55]. Complementary functional–structural and virtual-architecture models simulate how canopy traits influence foliar fungal epidemics, rain penetration, splash dispersal, and disease control in cultivar mixtures, illustrating why pathogen movement should be interpreted through 3D plant arrangement rather than solely through aggregate host resistance [17,21,56]. Although Septo3D predates current digital-twin terminology, this branch captures an essential principle: crop geometry can influence disease processes, and protection modelling should therefore incorporate architecture rather than rely only on aggregate plant descriptors.

The measurement-oriented disease literature points in the same direction, although with less uniform decision maturity. Broader disease-imaging reviews and architecture–pathology studies indicate that protection phenotyping needs to account for sensor-specific disease constraints and for the ways in which plant architecture modifies pest and pathogen attack. Recent 3D disease-severity studies, including stereo–thermal detection of diseased tomato plants, deep segmentation of grape foliar diseases, 3D imaging for soybean frogeye leaf spot assessment, and 3D convolutional assessment of Fusarium head blight in wheat, suggest that disease quantification is beginning to move from projected symptom area toward spatially richer representations [35,36,57,58]. This should not be read as a general claim that 3D always outperforms 2D disease imaging. Rather, the strongest case for 3D arises when symptoms are organ-localized, vertically distributed, occluded, or linked to canopy structure in a way that a projected image may miss.

Recent disease-oriented studies provide quantitative but heterogeneous evidence that 3D and depth traits can support symptom localization, severity scoring, or stress-state interpretation. In oil palm, terrestrial laser scanning characterized canopy changes associated with basal stem rot, and a combined frond-number/frond-angle/S200 model classified healthy versus unhealthy palms with 86.67% accuracy and four severity levels with 80% accuracy [14]. In sugar beet, a UGV multisensor platform converted 3D LiDAR point clouds into depth maps within an RGB–hyperspectral–thermal–LiDAR workflow, reporting strong plant–soil segmentation and moderate Cercospora severity scoring [37]; a complementary SfM-based multispectral 3D point-cloud study mapped Cercospora leaf spot within canopy space, reporting agreement with measured disease ratios and showing that vertical disease heterogeneity can be missed by conventional 2D spectral assessment [59]. In wheat, AR-glass imagery combined with Depth Anything V2 produced Fusarium-head-blight spike masks with high precision and IoU, and a fused colour index estimated severity with $R^{2}$ = 0.815 and RMSE = 8.91% [60]. These outputs are useful severity or localization signals, but most remain below treatment-decision evidence unless they are tied to fungicide timing, resistant genotype selection, field thresholds, or outcome validation.

A second group of studies reinforces the same point at canopy, multimodal, or temporal scales, but again the interpretation depends on comparator and task output. Wheat-rust monitoring linked 3D LiDAR intensity to modified Cobb-scale severity and yield loss across cultivar–stage combinations [61]; UAV hyperspectral potato-blackleg classification was improved by adding structural features, especially LiDAR-derived traits [62]; ground LiDAR combined with UAV multispectral features improved yellow-leaf-disease grading in areca palm by 23.94 percentage points over spectral-only features [63]; and hydroponic parsley root-rot detection indicated that morphometric information is useful, with the strongest performance arising from multimodal fusion [64]. These examples show that LiDAR or multimodal features can add value in some disease or stress settings, not that they provide a universal replacement for well-validated 2D or spectral disease workflows. Terrestrial LiDAR time-series analysis of maize drought also estimated plant height, plant area index, and projected leaf area against field measurements, thereby supporting stress-resilience interpretation before direct intervention rules are available [65]. Hyperspectral and deep-learning disease studies provide complementary symptom-detection evidence for yellow rust, viral disease, and explainable 3D/hyperspectral classification, although they should not be treated as complete 3D decision systems without trait-to-action linkage [66,67,68].

These examples support the disease and stress branches of Table 3 because they move beyond simple 2D symptom projection toward organ-, canopy-, or structure-aware evidence. However, the evidence is less uniform than in the spraying branch: some studies report disease or stress classification, others report structural proxies, and only a smaller subset connects those outputs to yield loss, severity scales, or management action. Their maturity should therefore be interpreted as L2–L3 unless severity classes, masks, or stress indicators are connected to treatment thresholds, fungicide timing, resistant genotype selection, or outcome feedback.

Weed-management evidence adds a complementary protection pathway in which depth, crop–weed geometry, or topology-aware perception defines the target for selective intervention. Earlier active-stereoscopy work on 3D weed images illustrates the historical boundary between depth-assisted crop–weed discrimination and later action-facing weeding systems [69]. Depth-camera reconstruction of weed-infested maize provides a measurement-layer example, linking Kinect-derived 3D point clouds and volumetric features to crop and weed biomass or density estimates without itself demonstrating herbicide actuation [70].

The more decision-facing evidence appears when target perception is linked to mechanical or chemical action. A mixed-autonomous robotic platform used RGB-D information and crop-position constraints to support intra-row and inter-row mechanical weed removal, reporting high plant and weed identification rates while remaining a prototype-stage system rather than a mature autonomous field-deployment platform [15]. Other robotic studies show how crop–weed classification and stereo or 3D tracking can be converted into nozzle trajectories, predictive control, and selective in-row weed treatment [71,72]. Recent maize–weed detection work further links lightweight perception to weed-pressure decision support through a topology-reconstructed detector and a visual weed–crop competition index, while low-cost photogrammetric 3D weed modelling and high-speed crop–weed identification in lettuce provide additional target-perception evidence for selective weeding [73,74,75]. These studies strengthen the pest/weed and robotic-intervention rows of Table 3, but they should still be interpreted as target-modelling or action-chain evidence rather than proof of closed-loop 3D crop-protection twins.

### 5.3. Task-Level Synthesis Across Protection Applications

Taken together, these studies support a tiered interpretation rather than a single claim that 3D phenotyping improves all protection tasks. The strongest tier is direct intervention evidence, where 3D traits are translated into dose adjustment, deposition prediction, drift reduction, airflow control, or actuator-level spray variables and are compared with conventional practice or field-performance measures. A second, more heterogeneous tier consists of disease, stress, and architecture-aware modelling studies, where depth, LiDAR, or multimodal traits can improve severity localization, structural interpretation, or process representation, but often stop at masks, severity scores, proxy traits, or scenario simulations. A third emerging tier includes pest, weed, scouting, IPM, and breeding links, where 3D information may define targets or structural risk traits but usually lacks economic thresholds, treatment timing, or outcome feedback. This evidence gradient supports a cautious conclusion: many studies demonstrate trait-readiness or component-level decision support, while fewer demonstrate validated intervention decisions, and still fewer approach closed-loop protection systems. Table 3 converts this gradient into task-oriented review categories.

The task mapping in Table 3 also clarifies why digital-twin terminology requires caution. A system may reconstruct canopy structure or estimate a disease-associated trait but still remain a measurement or monitoring system if it does not update, simulate, predict, recommend, or learn from intervention outcomes. The following maturity ladder therefore evaluates digital-twin claims according to demonstrated coupling and decision role rather than terminology.

**Table 3:** Protection-decision pathways enabled by 3D traits. Rows are organized by the decision unit changed by 3D information rather than by sensor type.

Decision Pathway	3D Trait or Representation	How 3D Information Changes Protection Reasoning	Maturity Signal	Representative Evidence and Interpretation
Dose adjustment for 3D crops	Canopy volume, leaf area index, target density, canopy height, and row geometry.	Converts target geometry into pesticide dose, spray-volume, airflow, or prescription-map logic.	Strongest when dose reduction is paired with coverage, deposition, efficacy, disease control, or drift validation.	DOSA3D, vineyard/orchard VRA, PWM and profile-variable spraying, laser- or LiDAR-guided sprayers, RGB-D vineyard flow control, UAV/SfM canopy guidance, and intelligent disease or pest spraying form the clearest geometry-to-intervention evidence stream [12,13,20,22,23,24,25,26,27,29,39,40,45,46,48,49,53,54,76,77,78].
Disease severity assessment and symptom localization	Organ surfaces, depth maps, multispectral 3D point clouds, LiDAR intensity, spike or leaf masks, and lesion position in canopy context.	Can improve severity localization when disease expression is organ-specific, vertically distributed, or occluded, but does not automatically outperform 2D workflows in all settings.	Disease class, severity score, infected-organ mask, or spatial disease distribution; higher maturity requires thresholds, treatment timing, genotype decision, or outcome validation.	Stereo–thermal tomato detection, grape disease segmentation, soybean frogeye 3D imaging, oil-palm basal-stem-rot metrics, sugar-beet LiDAR/depth and Cercospora 3D mapping, wheat FHB depth assessment, wheat rust LiDAR, potato-blackleg structural features, areca YLD LiDAR–multispectral grading, and parsley root-rot fusion support symptom and severity evidence, but usually remain below closed-loop decision maturity [14,35,36,37,57,58,59,60,61,62,63,64,66,67,68].
Architecture-aware disease-process modelling	Virtual plants, canopy architecture, organ position, leaf angle, and microclimate-relevant structure.	Represents how crop architecture modifies infection, dispersal, splash transport, canopy microclimate, and epidemic progress.	Scenario analysis, risk state, or model-predicted epidemic class; stronger when calibrated with field observations.	Septo3D, coupled virtual wheat–Septoria modelling, functional–structural fungal-epidemic models, and virtual-architecture splash-dispersal models provide the conceptual bridge from structure to disease-process reasoning [16,17,21,55,56].
Pest, weed, or stress scouting and threshold linkage	Canopy structure, plant height, gap fraction, spectral–structural stress proxies, affected-plant distribution, and crop/weed geometry.	Uses structural or depth-aware crop state to interpret pest habitat, weed competition, drought dynamics, or economic-injury signals, but often remains target or risk mapping rather than threshold-based intervention.	Scouting priority, risk map, treatment-threshold trigger, or robotic weeding target; pest-specific economic-threshold evidence remains limited.	Areca YLD grading, root-rot fusion, temporal maize drought LiDAR, soybean stress point-cloud classification, weedy-field crop recognition, photogrammetric weed modelling, topology-aware maize–weed detection, RGB-D robotic targeting, 3D-tracking robotic weeding, and planthopper-resistance evaluation show a growing but heterogeneous scouting-to-threshold pathway [15,63,64,65,72,73,74,75,79,80,81].
Spray deposition, off-target loss, and robotic intervention	Target surface, occlusion, canopy porosity, voxelized canopy structure, row geometry, and robot-localized weed or pest targets.	Aligns intervention hardware with target geometry and exposure, reducing under-coverage, drift, and unnecessary input.	Deposition uniformity, penetration, off-target-loss reduction, prescription map, PWM command, airflow command, nozzle trajectory, or robot action.	Voxel canopy deposition models, disease-and-canopy-volume dosage optimization, LiDAR/UAV/orchard canopy-volume spraying, porosity-informed penetration models, air-assisted deposition, real-time air/liquid control, drift studies, and robotic weed recognition or removal provide field-facing evidence, but most remain open-loop components [15,18,19,22,23,24,25,26,27,41,44,45,46,47,48,49,50,51,52,71,72,76,77,78,80,82].
Protection-oriented breeding and ideotype selection	Plant height, organ arrangement, canopy compactness, architecture traits, and genotype-specific 3D time series.	Selects structural traits that influence disease escape, sprayability, microclimate, pest habitat, or stress resilience.	Trait ranking, genotype selection, or ideotype design; generally upstream of immediate management decisions.	Field LiDAR, CropQuant-3D, organ-level point-cloud methods, drought time-series LiDAR, horticultural canopy-density estimation, and FSPM bridges provide the infrastructure for protection-oriented ideotype design [4,7,10,11,28,65].

Part 1 covers pathways where 3D traits alter dose, severity assessment, or disease-process interpretation. Rows should be read as evidence pathways, not as claims that 3D is always superior to 2D or spectral alternatives. Review weight increases from trait measurement to comparator-based task improvement, field validation, intervention output, and outcome feedback.

## 6. Digital Twins: Maturity Rather than Terminology

### Definition Boundary and Maturity Criteria

Digital-twin terminology is useful only when it clarifies the level of data–model–decision coupling. Following the broader smart-farming and agricultural digital-twin literature [9], this review distinguishes static 3D models from digital twins. One limitation of many digital-twin claims in this area is that they do not adequately distinguish visualization or offline simulation from systems that are synchronized, predictive, action-oriented, and updated by field outcomes. A 3D visualization, crop dashboard, or offline simulation should therefore not be labelled a digital twin unless it includes explicit data–model coupling and some form of updating, prediction, or decision feedback. In the ladder below, each level is assigned according to the highest function demonstrated by the evidence, not according to the sophistication of the sensor, reconstruction algorithm, or terminology used by the original study.

This review proposes a maturity ladder for 3D crop-protection twins. Level 0 denotes a 3D model only. Level 1 is a geometric twin that represents crop structure in metric form. Level 2 is a phenotypic twin that extracts and validates traits. Level 3 is a functional twin that couples traits or geometry to growth, physiology, microclimate, disease, stress, or spray processes. Level 4 is a protection twin that predicts a protection-relevant risk, dose, deposition, intervention response, or timing output. Level 5 is a decision twin that recommends or executes actions and updates from field outcomes. The most common boundary cases are handled conservatively: L2 requires validated traits but no explicit process model; L3 requires process coupling but no action recommendation; L4 requires a protection output or recommendation that can be evaluated; and L5 requires outcome recording plus model, rule, or prescription updating. Under this ladder, FSPM-based studies and agricultural digital-twin frameworks provide important bridges [10,11,83], but they should be classified according to demonstrated coupling, validation, decision role, and feedback status. Table 4 formalizes the ladder used in this review.

**Table 4:** Digital-twin maturity ladder used to classify 3D crop-protection studies. The ladder is evidence-based: systems are assigned to the highest level that is demonstrated, not to the level implied by terminology.

Level	Name	Minimum Qualification	Boundary or Validation Requirement	How Evidence Is Interpreted
L0	3D model only	A static or reconstructed 3D representation is produced.	Does not qualify for L1–L5 if traits are not validated, no time comparison is possible, and no protection output is extracted.	High-fidelity NeRF, 3DGS, mesh, or point-cloud outputs remain L0 when evaluated mainly by visual or geometric fidelity [3,31,33].
L1	Geometric twin	Crop structure is represented metrically and can be compared across plants, plots, or time points.	Requires scale, registration, or repeatability sufficient for structural comparison; without validated biological traits it does not reach L2.	Field LiDAR, CropQuant-3D, and point-cloud datasets usually support L1 when their main contribution is structural reconstruction or segmentation infrastructure [4,5,6,8].
L2	Phenotypic twin	3D geometry is converted into validated traits such as height, leaf angle, organ count, canopy volume, lesion-relevant surfaces, LiDAR intensity, or depth-derived disease features.	Requires validation against measurements, labels, severity classes, or field observations; without explicit process coupling or decision rules it remains L2.	Organ segmentation, canopy trait extraction, drought LiDAR, disease-associated structure, depth-assisted disease severity, stress point clouds, topology-aware weed pressure, and pest-resistance traits occupy L2 when not embedded in process models or decision rules [7,14,37,58,60,61,63,64,65,75,79,81].
L3	Functional twin	Traits or geometry are coupled to growth, physiology, microclimate, epidemiology, spray transport, or other process models.	Requires an explicit process model or simulation state; without a protection output, dose, risk, timing, or recommendation it does not reach L4.	FSPM, Septo3D-like disease coupling, coupled virtual wheat–Septoria modelling, fungal epidemic simulation, virtual splash-dispersal studies, and canopy models for disease management approach L3 because they connect structure to functional or pathogen processes [10,11,16,17,21,55,56,83,84].
L4	Protection twin	The coupled system predicts protection-relevant risk, severity, dose, deposition, intervention response, or treatment timing.	Requires a decision-facing output that can be compared with field performance, baseline practice, or intervention outcomes; without outcome updating it does not reach L5.	Dose adjustment, deposition prediction, drift reduction, disease-aware VRA, canopy-volume spraying, SfM aerial-spraying guidance, porosity-to-penetration modelling, real-time dosage control, prescription maps, disease-warning placement, and grey-mould morphology descriptors provide partial L4 components; they should not be generalized into synchronized twins without updating and feedback [12,13,18,19,22,23,24,25,26,27,40,41,44,45,46,47,48,49,50,51,52,53,54,77,78,82,85,86].
L5	Decision twin	The system recommends or executes actions, records field outcomes, and updates model states, rules, or prescriptions through feedback.	Requires a closed loop: observation, recommendation or action, outcome recording, and update of the model, rule, threshold, or prescription.	IPM decision-support and robotic pest- or weed-control architectures show partial L5 requirements, but broadly validated closed-loop 3D crop-protection twins remain rare [15,80].

Part 1 separates reconstruction and trait-readiness from functional or decision coupling. The L2–L3 boundary is process coupling: validated traits alone are L2 unless they are embedded in a biological, physical, epidemiological, or spray-process model. The key distinction between L4 and L5 is feedback. L4 may predict, prescribe, or control; L5 additionally requires field-outcome recording and model, rule, threshold, or prescription updating.

This maturity perspective helps to avoid terminological inflation. For example, high-fidelity wheat reconstruction using 3DGS or NeRF may represent an advanced L0–L1 output if it lacks validated traits or feedback [3]. CropQuant-3D and organ-level point-cloud segmentation can support L1–L2 phenotyping layers [4,7]. Septo3D-like disease coupling, coupled virtual wheat–Septoria modelling, and FSPM frameworks approach L3 because they connect structure to function or disease processes [10,16,17,55]. DOSA3D and VRA systems are retained as L4-type components when canopy state changes dose, deposition prediction, drift reduction, risk descriptors, or flow control, including recent examples where SfM canopy height and volume agree strongly with LiDAR for UASS parameter guidance, LiDAR-derived optical porosity supports droplet-penetration modelling, and LiDAR canopy volume drives real-time orchard dosage control [12,13,18,19,20,39,40,41,44,45,46,47,48,77,78]. Digital-twin-labelled morphology studies can contribute descriptors for risk evaluation, such as bunch architecture in relation to grey mould, but they should still be interpreted through the same evidence thresholds [86]. These examples do not establish broadly synchronized, field-validated, closed-loop crop-protection decision twins unless the outcome-updating requirement is demonstrated. Figure 3 summarizes this maturity-positioning logic by showing that technical representation must cross an evidence threshold before it can support protection outputs, decision rules, and outcome feedback.

## 7. Current Evidence Gap: The Missing Protection Feedback Loop

The current evidence can be summarized as a mismatch between measurement maturity and decision maturity. On the measurement side, 3D reconstruction, point-cloud datasets, segmentation benchmarks, and neural representations are advancing rapidly [2,5,6,8]. On the decision side, the strongest active links currently involve geometry-to-dose adjustment, deposition prediction, architecture-aware disease modelling, canopy-sensed drift reduction, temporal stress phenotyping, depth/3D-assisted disease-severity measurement, and selective weed-target perception [12,13,16,17,18,19,20,21,23,24,39,40,41,44,45,46,47,48,51,52,56,57,61,65,73,74]. Digital-twin and FSPM papers provide a conceptual bridge; nevertheless, many studies remain at the component level unless sensor updating, model calibration, prediction, action recommendation, and decision feedback are demonstrated [9,10,11,86].

The practical gap is therefore not a shortage of 3D measurements, but a weak chain from measurement to protection consequence. This weakness can be separated into three linked evidence gaps. The first is a trait-validity gap: 3D traits must represent biologically or physically meaningful protection processes rather than visual structure alone. The second is a decision-utility gap: those traits must improve a severity score, risk state, dose variable, treatment timing, scouting priority, or breeding target relative to 2D, spectral, scalar, or conventional baselines. The third is a feedback gap: the system must record field outcomes and use them to update model states, intervention rules, or recommendations. Disease and stress studies increasingly report severity classes, lesion masks, canopy-state indicators, or temporal structural traits, but these remain L2–L3 advances unless they support thresholds, treatment timing, genotype selection, or outcome-updated recommendations. Spraying and deposition studies are closer to decision use because canopy traits are translated into dose, deposition, drift, airflow, or prescription-map outputs, and because several field systems evaluate input reduction or off-target loss. Even so, most of these studies still validate open-loop components rather than feedback-updated protection twins.

A practical feedback loop should be reported as a minimum sequence rather than as a general aspiration: (i) acquire synchronized crop geometry and supporting sensor data; (ii) extract a named 3D trait with units, uncertainty, coordinate frame, timestamp, sensor provenance, and calibration status; (iii) compare the trait with a baseline, threshold, or decision rule; (iv) issue a recommendation, prescription, robotic action, or breeding/scouting priority; (v) record the field outcome, such as deposition, efficacy, disease progress, off-target loss, yield effect, or scouting confirmation; and (vi) update the model state, threshold, prescription, or rule. This sequence converts the feedback requirement into an auditable reporting item and clarifies why a dashboard or one-time simulation is not yet a decision twin.

This gap structure suggests a shift in how the evidence should be read: the central question is no longer whether crops can be reconstructed in 3D, but how 3D traits change protection outcomes. The most useful evidence therefore reports not only reconstruction accuracy, but also biological validity, protection-task relevance, comparison with 2D baselines, intervention thresholds, temporal update frequency, field robustness, and outcome feedback. A useful minimum reporting unit is the complete pathway from crop and protection target to 3D trait, comparator, task output, action boundary, validation context, and feedback status. For disease and pest systems, the key issue is whether 3D traits are linked to pathogen or pest ecology rather than to visual symptoms alone. For spraying, it is whether 3D geometry is linked to deposition, efficacy, drift, and dose reduction. For breeding, it is whether 3D architecture is linked to protection-oriented ideotypes and genotype-by-environment responses.

Thus, Table 2, Table 3 and Table 4 can be read sequentially. Table 2 defines the measurement options, Table 3 defines the protection uses, and Table 4 defines the minimum evidence required before a 3D-enabled system can be interpreted as a digital twin. Table 5 then converts weak layer connections into an agenda: benchmark labels, field transfer, trait validation, decision comparison, semantic-temporal understanding, process coupling, feedback validation, and interoperability. The remaining barriers arise where these layers fail to connect.

## 8. Bottlenecks and Research Agenda

Several bottlenecks recur across the evidence reviewed. First, protection labels are sparse in many 3D datasets. Such datasets may support segmentation or reconstruction, but not disease, pest, stress, or spray-decision tasks; they should therefore be treated as reproducibility and benchmarking infrastructure unless protection outcomes are explicitly labelled. Second, field transfer remains difficult because illumination, occlusion, wind, growth stage, and canopy density affect both reconstruction and trait extraction. Field LiDAR studies can improve plot-scale structural robustness [34], but they often remain focused on height or biomass rather than protection decisions.

The more decision-facing bottlenecks concern validation, feedback, and interoperability. Digital-twin claims often lack explicit synchronization and feedback, and only a subset of studies reports whether 3D information improves decisions relative to simpler 2D, spectral, or scalar baselines. The areca YLD study, where LiDAR added 23.94 percentage points over spectral-only features, and spraying studies in which canopy-aware control reduced input use while maintaining deposition or efficacy, provide useful comparator-based examples [13,63,77]. However, such comparisons are not yet routine across disease, pest, stress, breeding, and robotic-intervention studies. Interoperability among sensing, reconstruction, trait extraction, process modelling, and decision systems also remains underdeveloped.

The evidence reviewed therefore points to six protection-oriented priorities: (i) benchmark datasets with protection labels and temporal metadata; (ii) organ- and canopy-level traits explicitly tied to disease, pest, stress, or spray mechanisms, building on organ-resolved point-cloud segmentation evidence [38]; (iii) field trials that evaluate intervention outcomes rather than reconstruction metrics alone; (iv) FSPM and disease-process models updated by sensor-derived 3D traits; (v) treatment-threshold and intervention-timing studies that connect sensor-derived states to fungicide, insecticide, scouting, or robotic action; and (vi) digital-twin architectures that report their maturity level, synchronization frequency, action boundary, outcome log, and update rule. Several of these priorities remain target gaps rather than well-supported claims.

For reporting, a useful minimum unit is a complete protection pathway rather than an isolated accuracy value. The relevant information is the crop and protection target, the 3D trait or semantic object measured, the baseline it improves upon, the decision variable affected, the field condition under which it was validated, and whether outcomes were used to update the model or recommendation. Practical interoperability begins with standard metadata for point clouds and derived traits: coordinate reference, scale, timestamp, growth stage, sensor type, calibration record, platform trajectory or view geometry, trait units, uncertainty, occlusion status, and links to intervention or outcome records. Such a reporting standard would make it easier for readers to compare disease, pest, stress, spraying, and breeding studies on the same maturity scale. Table 5 summarizes these bottlenecks and converts them into a future research agenda. Taken together, these directions would help move 3D crop phenotyping from measurement capability toward evidence-based decision support for sustainable crop protection.

**Table 5:** Decision-facing bottlenecks and research agenda for 3D crop phenotyping in crop protection. The agenda is organized around the missing links that prevent 3D measurements from becoming validated protection decisions.

Bottleneck	Observed Pattern	Why It Limits Protection Value	Research Priority	Evidence Needed
Protection labels and task-specific benchmarks	Many 3D datasets support reconstruction or segmentation but not disease, pest, stress, spray-accessibility, treatment-threshold, or intervention-efficacy labels.	Without protection labels, benchmarks measure technical reproducibility rather than agronomic utility.	Build open 3D/4D datasets with protection labels, temporal metadata, field context, and task-specific evaluation splits.	Dataset and benchmark studies extending Crops3D, Pheno4D, and Wheat3D PartNet toward protection tasks [5,6,8].
Field robustness and transferability	Lab, greenhouse, or single-platform success often does not demonstrate multi-season, multi-crop, or cross-field reliability.	Protection systems must operate under wind, illumination change, occlusion, dense canopies, growth-stage variation, and platform constraints.	Report field protocols, uncertainty, failure modes, cross-season tests, and crop-specific deployment limits.	Plot- and field-scale LiDAR or platform studies with explicit transfer analysis [4,34].
Trait-to-decision gap	Many papers stop at plant height, volume, organ segmentation, point-cloud quality, or visual reconstruction.	3D traits matter for crop protection only when they alter severity assessment, risk classification, dose, timing, scouting, robotic action, or breeding choice.	Link traits to decision units such as disease thresholds, economic injury levels, prescription maps, spray deposition, intervention outcomes, or genotype selection.	Direct application studies across dose adjustment, VRA, deposition/drift prediction, prescription mapping, disease severity, stress phenotyping, weed targeting, pest-resistance evaluation, and timing models [12,13,18,23,24,25,44,45,46,47,48,49,50,51,52,57,58,61,65,66,67,68,73,74,79,81,82].
Semantic and temporal organ-level understanding	Whole-canopy point clouds may lack organ identity, lesion localization, pest-habitat representation, spray-surface exposure, or temporal correspondence.	Protection targets are often organs, lesions, pest habitats, and exposed surfaces rather than whole-plant geometry.	Develop organ-, lesion-, target-, and time-aware 3D segmentation and tracking pipelines that preserve biological meaning.	Organ-resolved point-cloud segmentation, annotated part-segmentation datasets, and depth-assisted disease studies evaluated against protection labels [7,8,38,60].
Multimodal and process coupling	Geometry-only systems may be descriptive, whereas purely data-driven models may lack mechanistic transferability.	Disease, pest, stress, and spray risks depend on geometry plus spectral, thermal, weather, microclimate, physiological, and pathogen processes.	Fuse 3D structure with spectral/thermal sensing, weather, FSPM, epidemiological, spray-transport, and physiological models.	Multisensor disease platforms, temporal LiDAR stress phenotyping, FSPM bridges, and architecture-aware disease models [10,16,37,56,65].
Digital-twin validation and feedback	Many twin claims remain conceptual, visualization-based, or model-only; field feedback loops are rarely demonstrated.	Digital twins require synchronization, prediction, decision rules, and outcome feedback, not only 3D scenes or dashboards.	Report observation frequency, action boundary, outcome variables, update target, and whether field outcomes changed the model, threshold, rule, or prescription.	L3–L5 studies that connect FSPM, disease risk, spraying, IPM decision support, and robotic control [9,11].
Interoperability and operational adoption	Pipelines often use incompatible formats, sensor-specific traits, isolated algorithms, or costly deployment assumptions.	Scalable protection tools must integrate sensors, traits, models, prescriptions, machinery, and farm decision systems.	Standardize point-cloud and trait metadata, coordinate frames, timestamps, calibration provenance, trait units, uncertainty, API handoffs, and links between prescription outputs and outcome logs.	Comparative deployment studies, precision-spraying systems with canopy-sensed control, and interoperable smart-farming architectures [9,45].

Part 1 identifies gaps in labels, field transfer, trait-to-decision validation, and semantic-temporal representation. The agenda is decision-facing: reconstruction, segmentation, and modelling advances are evaluated by their contribution to validated prediction, intervention, or feedback.

## 9. Conclusions

This review has argued that 3D crop phenotyping should be evaluated by the protection function it demonstrates, not by reconstruction novelty alone. The field now has strong enabling technologies in LiDAR, point-cloud processing, annotated datasets, organ segmentation, neural reconstruction, FSPM, architecture-aware disease modelling, canopy-sensed variable-rate spraying, and digital-twin frameworks. The evidence reviewed here is most convincing when reconstructed geometry is converted into validated traits, linked to protection processes, compared with simpler baselines, embedded in decision rules, and, at the highest maturity level, updated from field outcomes.

The evidence base is expanding rapidly, but its maturity remains uneven. Spraying and deposition studies provide the clearest decision-facing examples because canopy traits can be translated into dose adjustment, deposition prediction, drift reduction, airflow control, or prescription maps and can be evaluated against field-facing outcomes. Disease, pest, stress, and breeding studies provide important trait and modelling evidence, but they often require additional links to treatment thresholds, intervention timing, genotype selection, or feedback-updated recommendations. The maturity ladder developed in this review reduces the risk of overclaiming by distinguishing static 3D models from geometric, phenotypic, functional, protection, and decision twins. In this interpretation, high-fidelity 3D reconstruction, severity classification, depth-assisted symptom localization, canopy-volume dosing, sensor-based precise spraying, deposition prediction, resistance screening, and functional–structural simulation are successive evidence layers, not interchangeable proof of decision-twin maturity.

Further research is needed to establish whether 3D-derived traits improve disease thresholds, spray prescriptions, scouting priorities, breeding decisions, and feedback-updated recommendations under field conditions. A stronger evidence base will require protection-labelled 3D datasets, comparator-based field trials, uncertainty and transferability analysis, process-model coupling, intervention-outcome validation, and interoperable links between sensors, traits, models, prescriptions, machinery, and farm decision systems. Immediate progress can come from reporting standardized point-cloud and trait metadata, explicit action boundaries, outcome logs, and update rules. Under such conditions, 3D reconstruction and digital-twin design could become complementary components of predictive and actionable crop-protection support rather than parallel technology narratives.

## Data Availability

No new data were created or analyzed in this review. Data sharing is not applicable to this article.

## Abbreviations

The following abbreviations are used in this manuscript:

2D	Two-dimensional
3D	Three-dimensional
3DGS	3D Gaussian Splatting
FSPM	Functional–structural plant model
IPM	Integrated pest management
LiDAR	Light detection and ranging
MVS	Multi-view stereo
NeRF	Neural Radiance Field
PAI	Plant area index
PLA	Projected leaf area
RGB-D	Red–green–blue plus depth
SfM	Structure from motion
TLS	Terrestrial laser scanning
UAV	Unmanned aerial vehicle
VRA	Variable-rate application
